# Children's Hospital, Nottingham

**Published:** 1899-02-11

**Authors:** 


"An Old Pro" writes: After reading the letters in your issue of January 21st about the Nottingham Children's Hospital, I felt that I must write. That Miss Morrish had too much responsibility is admitted, though she may have over-estimated it, for though she was not often visited, doubtless the authorities knew exactly from the doctors how the case was going on. But it seems hardly fair for a new probationer to publish what was done when the hospital was in a strait for want of nurses, and especially so in the case of the matron, whose life and work in the hospital have been the admiration of so many nurses.

*** We have also received a letter signed by three house surgeons, W. F. Harvey, M.A., M.B., C.M., Wm. L. Garner, M.R.C.S., and J. H. Henderson, B.A., M.B., much to the same effect, and another comes from a gentleman who acted as such six years ago, and who has never visited the hospital since. We can only repeat that opinions of any kind cannot do away with stated facts into which no thorough investigation is made, and which are not emphatically met and denied by the authorities.

